# Characterization of Porous Scaffolds Fabricated by Joining Stacking Based Laser Micro-Spot Welding (JS-LMSW) for Tissue Engineering Applications

**DOI:** 10.3390/ma15010099

**Published:** 2021-12-23

**Authors:** Luis D. Cedeño-Viveros, Ciro A. Rodriguez, Victor Segura-Ibarra, Elisa Vázquez, Erika García-López

**Affiliations:** 1 Tecnológico de Monterrey, Escuela de Ingeniería y Ciencias, Ave. Eugenio Garza Sada 2501, Monterrey 64849, Mexico; luis.cedeno@tec.mx (L.D.C.-V.); ciro.rodriguez@tec.mx (C.A.R.); victor.segura@tec.mx (V.S.-I.); 2 Laboratorio Nacional de Manufactura Aditiva y Digital (MADiT), Apodaca 66629, Mexico

**Keywords:** JS-LMSW, laser micro-spot welding, laser micro-cutting, sheet lamination, additive manufacturing, bone scaffolds

## Abstract

A novel manufacturing approach was used to fabricate metallic scaffolds. A calibration of the laser cutting process was performed using the kerf width compensation in the calculations of the tool trajectory. Welding defects were studied through X-ray microtomography. Penetration depth and width resulted in relative errors of 9.4%, 1.0%, respectively. Microhardness was also measured, and the microstructure was studied in the base material. The microhardness values obtained were 400 HV, 237 HV, and 215 HV for the base material, HAZ, and fusion zone, respectively. No significant difference was found between the microhardness measurement along with different height positions of the scaffold. The scaffolds’ dimensions and porosity were measured, their internal architecture was observed with micro-computed tomography. The results indicated that geometries with dimensions under 500 µm with different shapes resulted in relative errors of ~2.7%. The fabricated scaffolds presented an average compressive modulus ~13.15 GPa, which is close to cortical bone properties. The proposed methodology showed a promising future in bone tissue engineering applications.

## 1. Introduction

Osteoporosis, trauma caused by fracture, debridement procedures done in bone infections, and resection of bone tumors are the main causes of human bone damage [1]. Despite bone tissue having a remarkable capacity to repair and heal itself after trauma and illness, critical-sized and large bone defects represent a challenge in today’s clinical practice due to bone repair which is limited by their dimension [1,2]. Bone tissue engineering provides a way to repair critical-sized defects by providing a three-dimensional structure called a scaffold [2,3]. Today, stainless steel and titanium alloys are the most common materials used in orthopedics load-bearing implants due to their good biocompatibility and their mechanical properties close to the natural bone or even higher [4]. For example, AISI302 stainless steel alloy has been reported as the first metallic alloy to fabricate orthopedic implants [5]. Some applications of AISI302 stainless steel are dental brackets [6] and orthodontic wires [7,8]. However, the AISI302 alloy is characterized by its good biocompatibility in the stomatognathic system’s tissues and its ability to achieve a wide range of mechanical properties by cold working and annealing with low manufacturing costs [9]. It has been replaced by AISI316L alloy due to its high corrosion resistance, which has allowed its application in bone plates, screws, and oral implants [6]. To the author’s best knowledge, there is only a study conducted by Cedeño-Viveros et al. [10] using AISI302 alloy in a sheet lamination process for potential applications of bone tissue engineering scaffolds.

Additive manufacturing (AM) has revolutionized the fabrication of complex structures for bone tissue engineering applications [11]. AM methods have the ability to customize the global shape and the internal architecture of the scaffold [4]. Laser powder bed fusion processes (LPBF) are commonly used for the fabrication of metallic bone scaffolds [4,11]. However, they have some typical disadvantages related to their high cost of implementation; they require extensive training, occupy large spaces, and are unable to produce high volumes [4,11]. A few works have been found in the literature regarding AISI316L scaffolds manufactured by AM (Table 1). These have reported a mechanical behavior similar to human cortical or cancellous bone mechanical properties. According to Hing et al. [12], there are several differences between cortical and cancellous bone in its stiffness, strength, and toughness. For example, cortical bone has a consistent density compared with cancellous bone, whose mechanical properties are dependent on its porosity and architecture.

Dewidar et al. [13] fabricated austenitic AISI 316L stainless steel scaffolds with a 50% porosity and with elastic modulus and compression strength close to the human cortical bone through the selective laser sintering process (SLS). Xie et al. [14] fabricated metal knee joints from coated stainless steel powder (i.e., 316L coated with ethylene-vinyl acetate resin) using the SLS process and obtaining parts with controlled porosity that can match the human cancellous bone properties. Čapek et al. [15] explored the mechanical properties of austenitic AISI316L stainless steel for producing highly porous scaffolds through selective laser melting (SLM) with mechanical properties close to the cancellous bone features. Vangapally et al. [16] studied different lattice designs with circular and square 2 mm pores and manufactured by binder jet additive manufacturing in AISI316 stainless steel for bone scaffolds applications.

In order to accomplish the scaffold design requirements, an evenly distributed pore and a highly controlled size promotes bone tissue ingrowth and facilitates the imitation of bone mechanical properties [17,18]. In this context, dimensional accuracy plays an important role in achieving the desired pore size with high morphological repeatability (i.e., shape, size, and distribution). In metal AM, small pore sizes have high difficulty in fabricating satisfactory samples of fine definition [11]. According to Garot et al. [4], the dimensional accuracies are ±300 µm and ±100 µm for LPBF.

Sheet lamination is an AM process that involves the joining and shaping of sheet feedstock [19]. In the last decades, sheet lamination techniques have been implemented to produce micro molds, micro-gears, and micro-electrodes for the electronic industry [20,21,22]. For example, Xu et al. [23] fabricated stainless steel (Cr18Ni9) micro mold cavities of 250 µm × 250 µm × 50 µm using a sheet lamination, which consisted in the laser cutting of metallic foils of 10 µm of thickness that were joined by micro-electric slip welding. The results showed horizontal and vertical size errors of 0.5 µm and 2 µm, respectively. Additionally, Xu et al. [20] fabricated Cu micro-electrodes with dimensional errors between 8.8 µm and 16.4 µm by cutting Cu foils of 100 µm of thickness using wire electrical discharge machining and joining them by vacuum pressure thermal diffusion welding.

Laser micro-cutting has demonstrated great potential to fabricate one of the most demanding medical devices, coronary stents, with high accuracy and high-quality surface finish [24]. Laser micro-welding is able to join all types of metallic materials and produce joints with high strengths, which are consistent with those of the base material [25]. Advantages of the laser micro-welding process are high precision due to the small size of the laser spot, non-contact to prevent contamination, and excellent weld quality with low heat input [26]. Applications of laser micro-welding include intraocular lenses, knee joint replacements, cochlear implants, and pacemakers [27,28]. Cedeño-Viveros et al. [10] studied sheet lamination using laser micro-cutting and laser micro-welding for bone scaffold applications with promising results. This investigation presents the study of bone tissue scaffolds fabricated through Joining Stacking based Laser Micro-Spot Welding (JS-LMSW) as a sheet lamination (additive manufacturing) process. Our methodology is based on the laser micro-cutting of AISI302 sheets, which are joined by laser micro-spot welds. In this paper, we present a calibration procedure implemented in the laser micro-cutting process to improve the dimensional accuracy of micro-shapes of the AISI302 scaffolds. Additionally, the mechanical behavior of the AISI 302 scaffolds fabricated by the proposed methodology was studied and compared with bone mechanical properties.

## 2. Materials and Methods

AISI302 (C0.05 Cr17.5 Ni8.1 Mn0.87 P0.003 S0.002 Si0.25 Mo0.19 wt%) sheets with 254 µm of thickness were used for experimental tests. The laser micro-cutting and microspot welding experiments were performed using a fiber laser machine (PRECO ST2000, Somerset, WI, USA) with a fiber core diameter of 50 µm. For the laser micro-cutting process, the focal plane was focused on half of the sheet’s thickness, and the standoff distance used was 254 µm for a measured spot size of 64 µm. For the laser micro-spot welding process, the standoff distance used was 14.5 mm to obtain a spot diameter of 143 µm on the top surface of the metallic sheets. The process parameters used for the micro-cutting and micro-spot welding are listed in Table 2.

### 2.1. Laser Micro-Cutting Specimen Calibration

**Figure 1:**
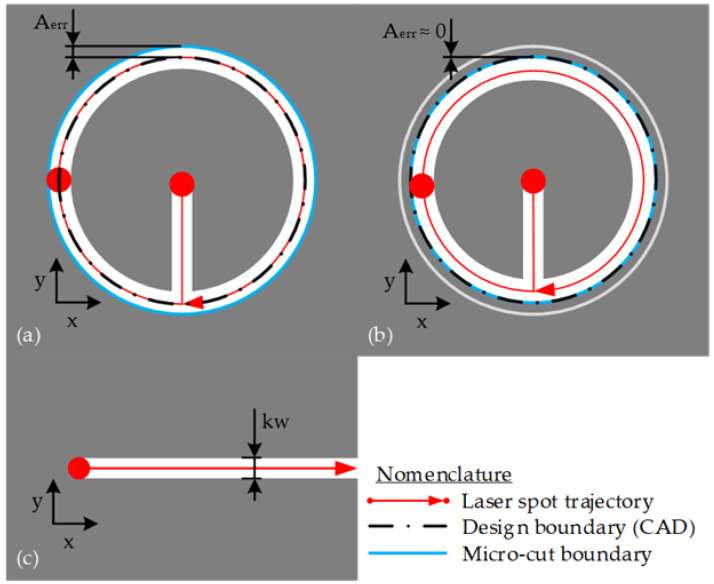
Laser micro-cutting trajectory (**a**) without kerf width compensation and (**b**) with kerf width compensation, and (**c**) kerf width (kw) measurement.

CAM software (Autodesk Fusion 360, California, USA) was used to obtain the toolpaths trajectories and the G-code to perform the laser cutting operations. Figure 1a,b shows the laser spot trajectory. The tool paths trajectories were calculated: without the kerf width compensation Figure 1a and with the kerf width compensation Figure 1b. Five lines of 10-mm length were laser cut in AISI302 shim with a thickness of 0.250 mm (Figure 1c). The measurements of the kerf width (kw) were performed in six points along the line on the top side using the Stereomicroscope V8 (Carl Zeiss, Jena, Germany) Figure 1c. These kerf width (kw) measurements were used to calculate the average kerf width to compensate for the tool’s trajectory in the laser cut.

**Figure 2:**
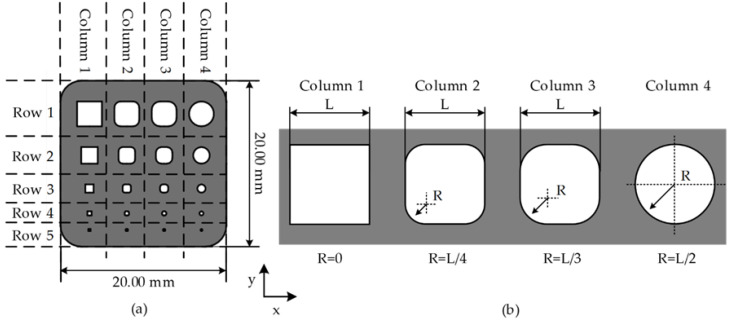
(**a**) Sample geometry design for laser micro-cutting and (**b**) row 1 with the design of shapes of each column.

The sample designed to study kerf width compensation was modeled using CAD software (Siemens NX10, Houston, TX, USA). Each sample consisted of an AISI302 sheet of 20 × 20 mm to obtain a rectangular array of five rows by four columns of different shapes. Each row consisted of a different length (L) in a range between 250 μm and 3000 μm, while each column had a different shape based on the corner radius (R). The corner radius (R) represents a portion of the length (L) (i.e., 0, L/4, L/3, L/2). A graphical view of the sample outline with dimensions is displayed in Figure 2a, and a detailed view of the different shapes of row 1 is displayed in Figure 2b. Additionally, Table 3 presents the nominal dimensions: length (L) and corner radius (R) of each row and column from the CAD model. For square and circle shapes, the corner radius (R) and the length dimension (L) were equal to 0, respectively. Six samples containing all shapes were produced in sheets: three samples were fabricated without the compensation of the kerf width, and three samples were fabricated with the compensation of the kerf width. After fabrication, images of the shapes were obtained using an inverted PMG3 optical microscope (Olympus, Tokyo, Japan). The shapes’ length (L) and corner radius (R) were measured in the ImageJ software (National Institute of Health, Maryland, USA). The relative error (${\mathrm{err}}_{R}$) was calculated using Equations (1) and (2). Where L and R correspond to the nominal values for the length and corner radius presented in Table 3. $L_{m}$ and $R_{m}$ are the measured length and corner radius in the fabricated shapes.

(1) $${\mathrm{err}}_{R}=\frac{\left|L-L_{m}\right|}{L}\;\times 100\%,$$

(2) $${\mathrm{err}}_{R}=\frac{\left|R-R_{m}\right|}{R}\times 100\%,$$

### 2.2. Scaffold Manufacture and Characterization

**Figure 3:**
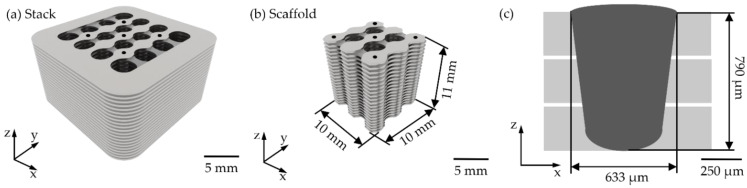
Designed scaffold (**a**) stack, (**b**) scaffold, and (**c**) laser micro-spot weld design.

The proposed scaffold consisted of cutting AISI302 20 × 20 mm sheets with a 0.254-mm thickness. Forty-three sheets were stacked using laser micro-spot welding. The scaffold was joined using the methodology explained in our previous work [10]. The final scaffold was cut from the stack using a wire electrical discharge machining (WEDM) machine. Six cubic scaffolds were created (10 × 10 × 11 mm). Figure 3 presents the scaffold designed with the established dimensions. Figure 3a shows the stack built after laser cutting and micro-spot welding, Figure 3b illustrates the scaffold after WEDM, and Figure 3c presents the micro-spot designed to achieve full penetration in accordance with the ISO13919-1 to join three AISI302 sheets and considering the gaps between sheets observed in the previous study [10]. An aspect ratio (d/w) of 1.25 was considered for spot weld designed to assure higher heat transference in the *Z*-axis and minimize mechanical distortions due to heat dissipation losses in the X and Y axes [29,30].

### 2.3. Scaffold Morphological Characterization

The scaffolds were scanned using micro-computed tomography (ZEISS Metrotom 1500, Jena, Germany) with an accelerating voltage of 125 kV and a beam current of 387 μA. The reconstructed images consisted of 468 × 372 × 441 voxels, with a voxel size of 38.72 μm^3^. Several images were captured on the XY, YZ, and XZ planes. The scaffolds’ porosity was determined by the Archimedes’ method in accordance with DIN 993-1, DIN 993-2, and DIN 623. The samples were weighed dry and in ethylic alcohol using an analytical scale (Mettler Toledo XPR205,Columbus, OH, USA) based on the methodology reported by Kumar et al. [27]. A scaffold was placed in polyester resin, and the cut was performed with a diamond blade. The sample was chemically attacked with Kallings solution to study the microstructure using an SEM microscope (Zeiss EVOMA25, Jena, Germany). Additionally, the chemical composition was obtained by an EDS analyzer (BRUKER X6/10, Billerica, MA, USA) in two different areas of the microstructure, identifying fusion zone (FZ), heat affected zone (HAZ), and the base material (BM). Additionally, a characterization was performed on the metallography image to identify defects such as pores, shrinkage, root cavities, excessive penetration, and excessive weld metal. These defects were observed in the scaffold cut in the XZ plane (See Figure 3).

### 2.4. Scaffold Mechanical Characterization

Five manufactured scaffolds were tested by compression evaluation in accordance with the ASTM E9 [28]. A universal testing machine (SHIMADZU AG-X, Kyoto, Japan) was used for the tests. The compression tests were performed with a constant crosshead speed of 1 mm/min with a 300 kN of load capacity at room temperature. The compressive modulus and compressive yield strength were calculated from the stress–strain curves. Images of the fractured samples were obtained with an SEM microscope (Zeiss EVOMA25, Jena, Germany) for failure analysis after compression tests.

The Vickers’s microhardness tests were executed according to the ASTM E384-17 [29] using a hardness testing machine (Zwick/Roell ZHVµ-S, Ulm, Germany). A Class B Vickers indenter certified (Euro Products Calibration Laboratory, Brieley Hill, England) in accordance with the ASTM E92-17. The indenter has a junction line size ≤ 0.05 mm and face angles of 136.03° and 135.88°.

The indentations were carried with a weight force of 25 g-F and the indentation time was 10 s, per point. Two evaluations of microhardness tests were performed. The first test was conducted in one micro spot weld. Microhardness was measured in a matrix of 11 × 3 points across the micro spot weld with a step distance of 0.254 × 0.125 mm between points (XZ-plane). In the second test, one scaffold was measured for microhardness in four micro–spot welds along the *Z*-axis of the scaffold. At each micro-spot, weld six indentations were executed: two in the base material (BM), two in the heat-affected zone (HAZ), and two in the fusion zone (FZ).

## 3. Results

### 3.1. Laser Micro-Cutting Specimen Calibration

**Figure 4:**
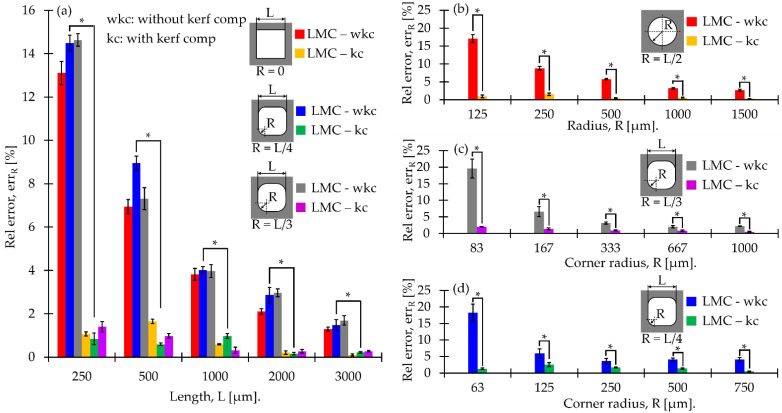
Relative error results from measured: (**a**) length (L) in pores with R = 0, L/4, L/3 and corner radius © in pores with (**b**) R = L/4, (**c**) R = L/3, and (**d**) R = L/2. Note: (wkc: without kerf compensation, kc: with kerf compensation, * *p* value ≤ 0.0001).

Figure 4 shows the relative error of length (Figure 4a), radius (Figure 4b), and corner radius of rounded squares (Figure 4c,d). In all figures, the relative error is significantly (*p*-value ≤ 0.0001) smaller when the kerf compensation is used. The dimensional analysis shows that the smallest feature (i.e., lower than 500 µm) increases the relative error considerably. Additionally, micro shapes (i.e., lower than 500 µm) with a kerf width compensation obtained a maximum relative error of 2.7% compared with non-compensated samples (i.e., the maximum relative error of 19.6%). While for macro shapes (i.e., higher than 500 µm), the maximum relative errors reported are 4.1% and 1.4% for non-compensated and compensated samples, respectively. This suggests that kerf width compensation improves dimensional accuracy when a cutting path is calculated.

**Figure 5:**
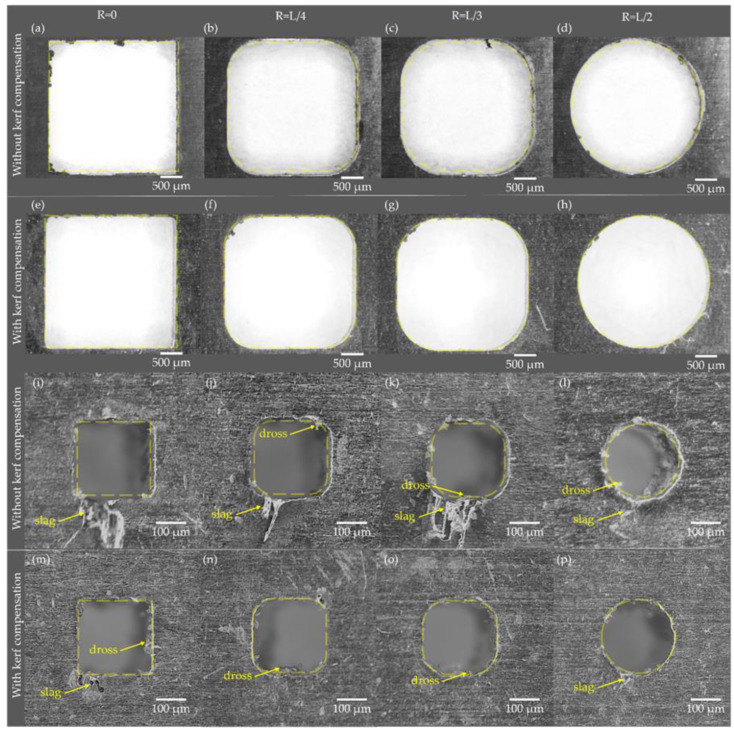
Macro shapes with a length of 3000 µm without compensation (**a**–**d**) and with compensation (**e**–**h**) and micro shapes corner radius with a length of 250 µm without compensation (**i**–**l**) and with compensation (**m**–**p**).

Figure 5a–h shows images obtained from laser cutting experiments with a length of 3000 µm without kerf compensation Figure 5a–d and with kerf compensation Figure 5e–h. Figure 5i–p presents the results of micro shapes with a length of 250 µm without kerf compensation Figure 5i–l and with kerf compensation Figure 5m–p. The yellow line indicates the shape profile designed using CAD software.

There is a greater difference between the yellow line and the manufactured shape when the length of the sample is 250 µm (Figure 5i–l) compared to 3000 µm of length (Figure 5a–d) when samples are non-compensated, which is related to the relative errors obtained before. Additionally, slag formation can be seen in 250 µm length non-compensated samples (Figure 5i–l). For micro shapes with a corner radius equal to 0, L/4, and L/3, the formation of dross (Figure 5m–o) and slag (Figure 5m,p) was observed.

### 3.2. Scaffold Morphological Characterization

**Figure 6:**
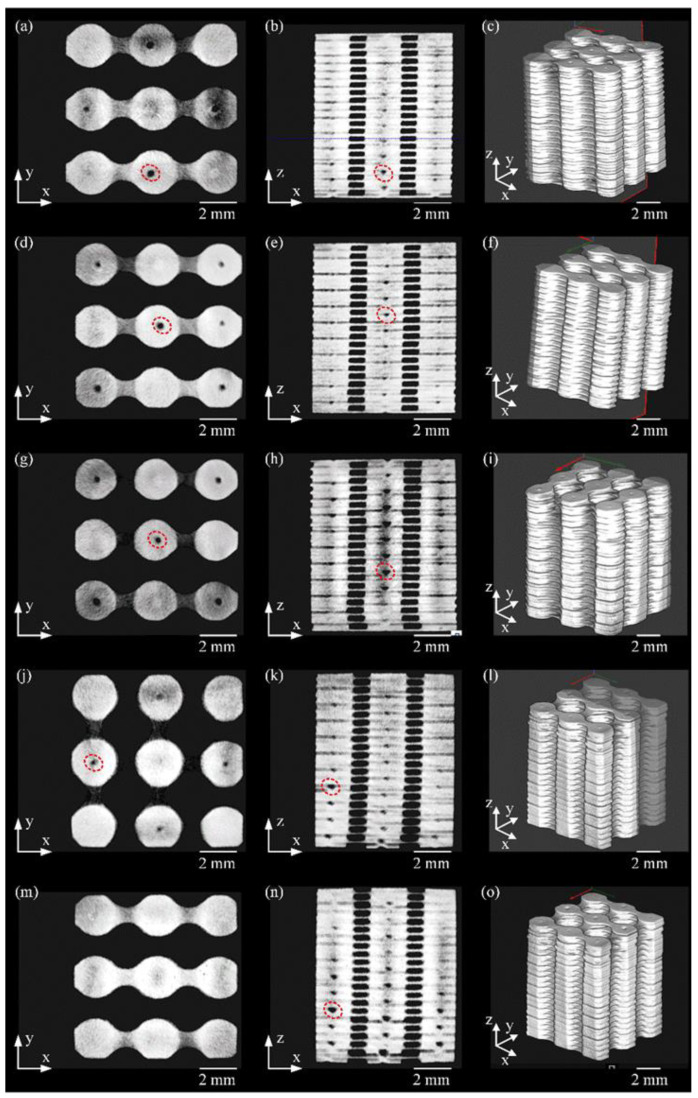
Pictures of the micro-CT analysis. Samples 1–5 (**a**,**d**,**g**,**j**,**m**) top, (**b**,**e**,**h**,**k**,**n**) lateral, and (**c**,**f**,**i**,**l**,**o**) isometric views.

**Figure 7:**
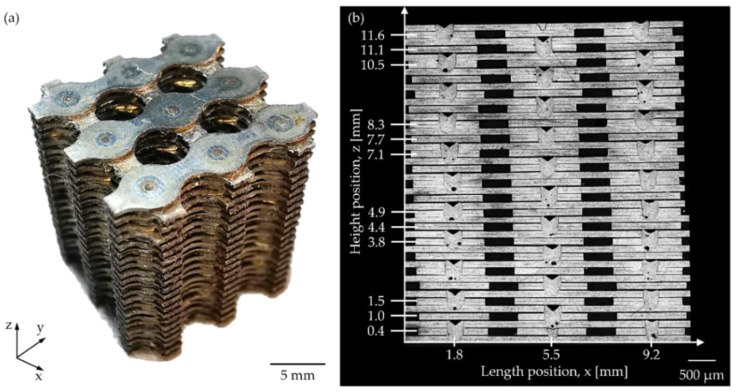
(**a**) Fabricated scaffold and (**b**) Metallography of the cross-section of the fabricated scaffold.

Microtomography images are shown in Figure 6. Black holes can be observed in Figure 6 (red oval) that indicate the presence of shrinkages. These shrinkages can also be identified in Figure 6a,b,d,e,g,h,j,k,n. No other defects were observed in the microtomography. Figure 6b,e,k,h,n shows micro-welding spots stacked in three columns, which suggests high repeatability in the position of the micro-welding spots. Figure 6c,f,i,l,o exhibit the scaffold reconstruction through X-ray tomography in isometric view. There is a high consistency of the final shape of the fabricated scaffolds. Figure 7a shows a scaffold manufactured. The scaffold’s final dimensions were 9.622 ± 0.013 mm in length and width, with a height of 11.564 ± 0.008 mm. The dimensional differences compared with design dimensions in the X and Y axis are attributed to the WEDM. The scaffolds resulted in a measured porosity of 46.5 ± 0.5% calculated by the Archimedes method, which is close to the designed porosity of 47% calculated by CAD software from the proposed design. Figure 7b displays the scaffold’s metallography used to observe the micro spot welds.

**Figure 8:**
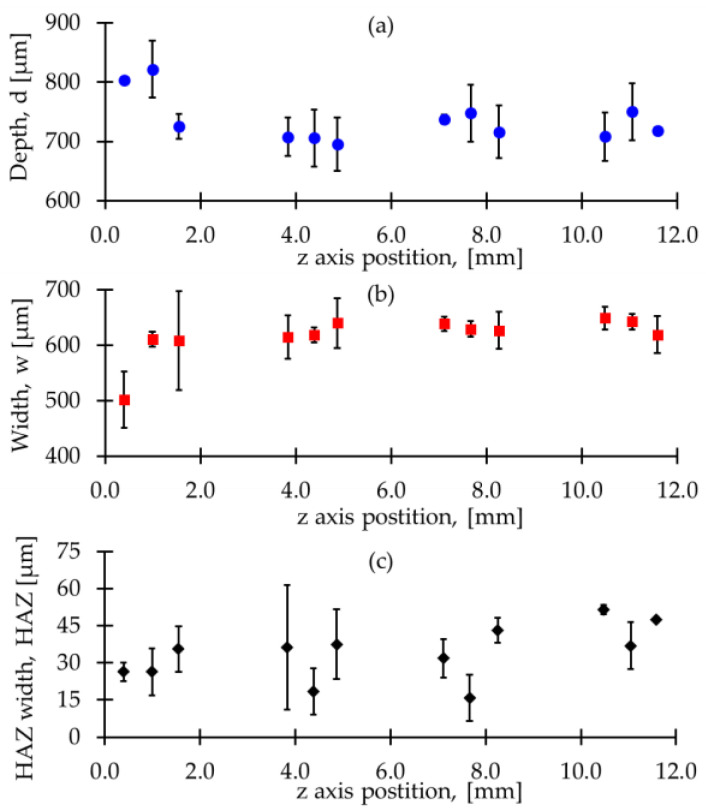
Results for the measured: (**a**) depth, (**b**) width, and (**c**) HAZ width of the micro spot welds.

The results of penetration depth, width, and HAZ width of micro-spot welds are shown in Figure 8. The penetration depth measurements (Figure 8a) decreased from 813 µm to values between 704 and 736 µm after 2 mm of the Z scaffold height. While the micro-spot welding width (Figure 8b) increased from 501 µm to values between 584 and 616 µm. HAZ measurements (Figure 8c) varied between 25 and 34 µm. These results demonstrate high repeatability in the micro-spot weld dimensions obtained in this works. Additionally, it is observed that the micro-spot welds under the Z height of 2 mm presented a fluted shape which is consistent with our previous study [10]. After the Z height of 2 mm, the micro-spot welds showed a parabolic shape; this change in the weld beads indicated a difference in the heat transfer phenomena [10,31] resulting from the stacking process.

**Figure 9:**
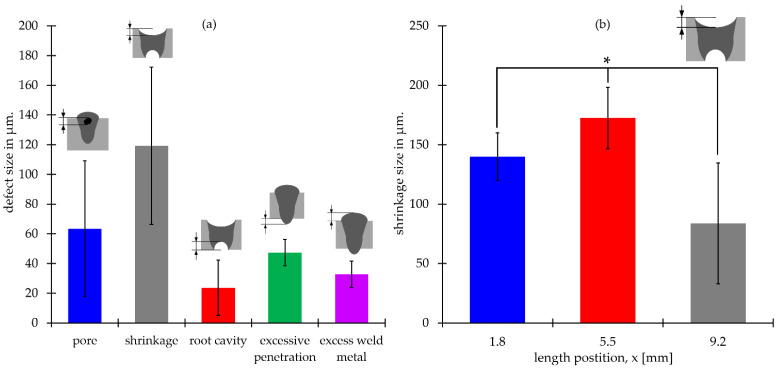
(**a**) Average size of the defects encountered in the micro-spot welds (**b**) measurements of the shrinkage defects in the micro-spot welds. * *p*-value ≤ 0.0001.

Welding defects were measured from metallography (Figure 7b). The results were plotted (Figure 9a) to evaluate micro spot quality and the shrinkages evaluation (Figure 9b). A total of 84 pores were found in the metallography section (Figure 7b). Figure 9 presents the pore’s size range of 52–75 µm; shrinkages in between 95 and 143 µm; root cavities in a range of 15-32 µm. Additionally, a single spot exhibited excessive penetration of ~24 µm. Every spot in the section presented excessive material welded with a size range of 29-37 µm. Due to shrinkages found in several micro-spot welds, they were evaluated along the *X*-axis (Figure 9b). The largest shrinkages were located in the center of the scaffold (x = 5.5 mm) and had an average size of 172 µm. The shrinkages located in the outer scaffold (x = 1.8, x = 9.2 mm) had an average size of 140 µm and 80 µm. The data reported in Figure 9b were analyzed using ANOVA (Analysis of Variance); our results indicated that with a confidence level of 95%, the differences between length positions are significant (*p*-value = 0.0001).

**Figure 10:**
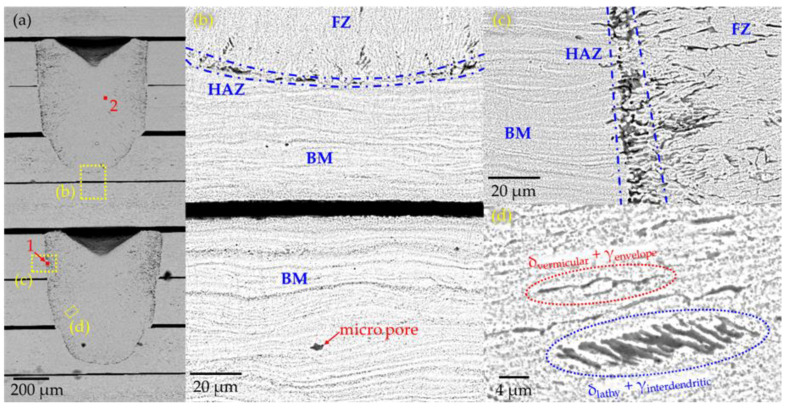
(**a**) Metallography of two micro spot welding (**b**) Lowest point of spot welding, (**c**) Lateral interface between spot welding and raw material (**d**) microstructure in the fusion zone.

Figure 10 presents the SEM micrographs from the fabricated scaffold (Figure 7a). Figure 10a shows dark areas near the HAZ compared with the central area of the spot weld, which indicates the reduction of the ferrite phase (δ) in the center of the spot weld. Several images were obtained at a different scale to observe the micro spot weld. Figure 10b illustrates the microstructure of two stacked sheets. The upper sheet presented the bottom interface between the fusion zone and the base material. In the fusion zone, epitaxial columnar dendritic growth was found, which is attributed to the solidification process. This columnar growth starts near the HAZ and goes to the central area of the spot weld, and in the base material, elongated and thin grains were present. Additionally, microporosities were found in the base material (Figure 10b). Figure 10c presents FZ, HAZ, and BM. The delimitation of HAZ was observed, and the lower HAZ of the weld was narrow compared with lateral HAZ. This indicates more heat diffusion in the X-Y axis than in the *Z*-axis. Additionally, Figure 10d presents a fusion zone composed of a vermicular ferrite (δ) phase wrapped in austenite (γ). Another identified structure was a lathy ferrite (δ) core with interdendritic austenite (γ) [32].

**Figure 11:**
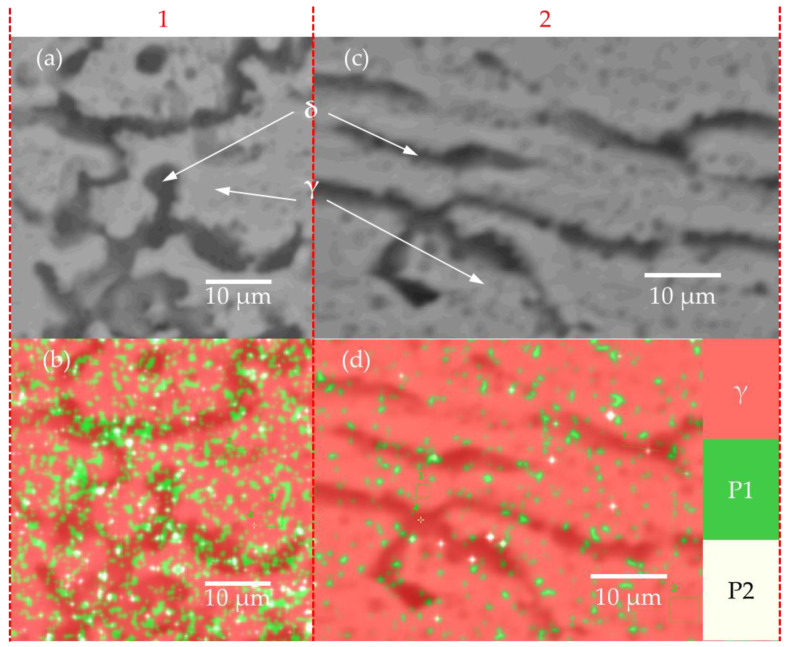
Color mapping in HAZ (1) and fusion zone (2): (**a**) SEM picture in HAZ (**b**) EDS mapping in HAZ (**c**) SEM picture in FZ, and (**d**) EDS mapping in FZ.

Figure 11 shows the results of a chemical analysis performed in EDS. Figure 11a,c indicates the HAZ and fusion zone, respectively. A color map of chemical composition (Figure 11b,d) was obtained for the same samples. Inside the austenite matrix (γ), the presence of precipitates with high Cr and Ni concentrations (marked in green color (P1) and white (P2)) was identified. The heat-affected zone presented a greater amount of these precipitates (i.e., 22.2% area) compared to the fusion zone (i.e., 5% area). Table 4 presents a summary of the phases (i.e., δ y γ) and precipitates (i.e., P1 y P2).

### 3.3. Scaffold Mechanical Characterization

**Figure 12:**
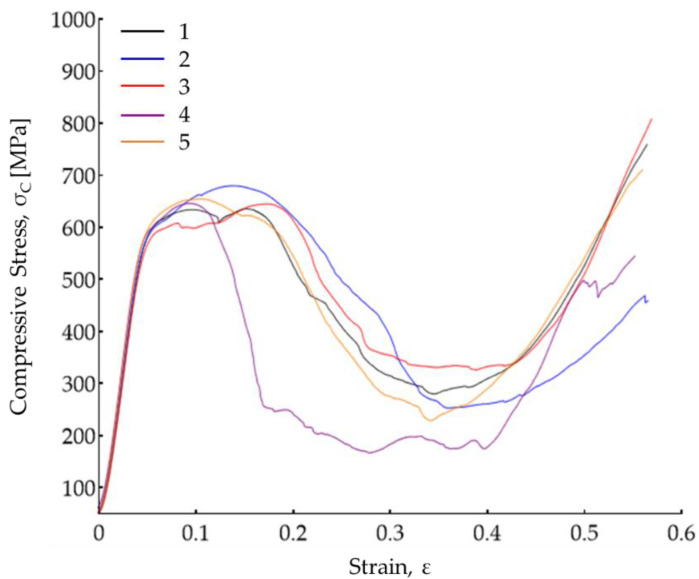
Compressive stress–strain curve of the compression test.

Figure 12 shows the stress–strain curves obtained in the compression tests, including raw data for five replicates. Four characteristic zones were observed for every fabricated scaffold: the elastic zone (ε = 0–0.05), yielding peak (ε = 0.05–0.20), stress plateau (ε = 0.20–0.40) and the densification zone (ε = 0.40–0.60). All five of the manufactured scaffolds presented a compression module between 13.03 and 13.26 GPa, a compressive stress range between 550 and 554 MPa, and a maximum compressive stress between 649 and 655 MPa.

**Figure 13:**
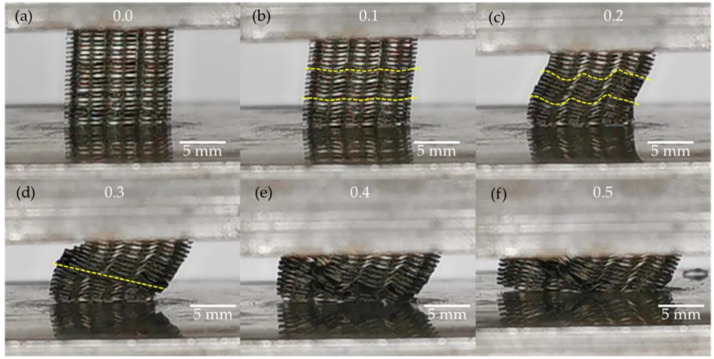
Compression test of the fifth sample at different strains (**a**) ε = 0, (**b**) ε = 0.1, (**c**) ε = 0.2, (**d**) ε = 0.3, (**e**) ε = 0.4, and (**f**) ε = 0.5.

**Figure 14:**
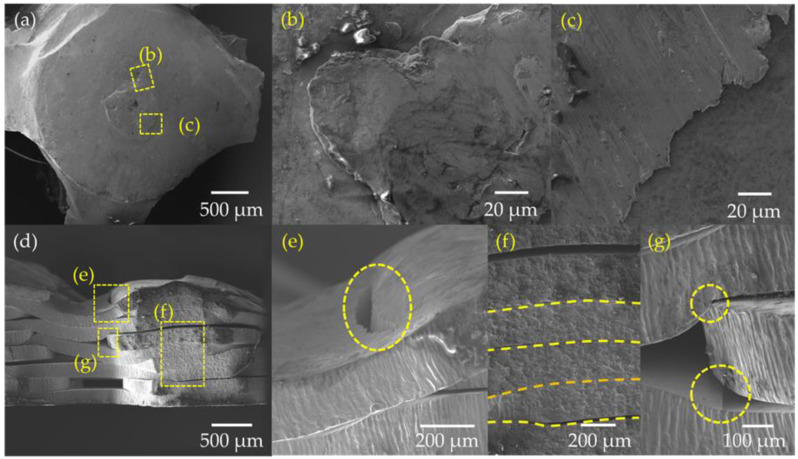
Fractographies of (**a**) micro-spot weld, (**b**) and (**c**) shear leaps of flat ductile fractures, (**d**) lateral view of the collapsed sheets, (**e**) crack due to collapse of sheets marked with a yellow circle, (**f**) close view of the thickness variations in the collapsed sheets, and (**g**) embedded zones with plastic deformation marked with yellow circles.

A graphic view of the compression test is shown in Figure 13. In the image corresponding to the compression test of the fifth sample (Figure 12), the yielding peak was reached between 0.1 and 0.2 of strain. Figure 13a presents the scaffold when the compression test starts. In Figure 13b, the sheets started to bend. This deformation continued in Figure 13c where some waves are observed and a warping effect on the scaffold structure. Sheets joined using micro spots started to slide between them after a strain of 0.3, see Figure 14d. Afterward, images showing deformation marked as 0.4 and 0.5 (Figure 13e,f) show the scaffold collapsing until it was completely compressed.

A fractography analysis was performed to study the scaffolding failure (Figure 14). A fractured micro-weld spot was found in Figure 14a. On the left side, plastic deformation was observed in the metal sheet as a result of the compression force between the stacked sheets. The fractured spot weld presented a shear leaps structure that indicates a flat and ductile fracture [33]; see Figure 14b. This was consistent with the slippage of the sheets that was observed during the compression test, see Figure 13c. Regarding the lateral view of the scaffold (Figure 14d), plastic deformation was observed in the sheets. This suggests that the slip between the sheets exceeds the elastic limit and causes the sheets to bend. Due to compressive loads, a crack caused by collapse was observed between the stacked sheets; see Figure 14e. It is also possible to notice a difference in the thickness of the metal sheets located in the center of the scaffold, see Figure 14f. The zones where the edges of a central sheet were embedded and produced cuts and plastic deformations in the surrounding sheets were identified with yellow circles, as shown in Figure 14g.

**Figure 15:**
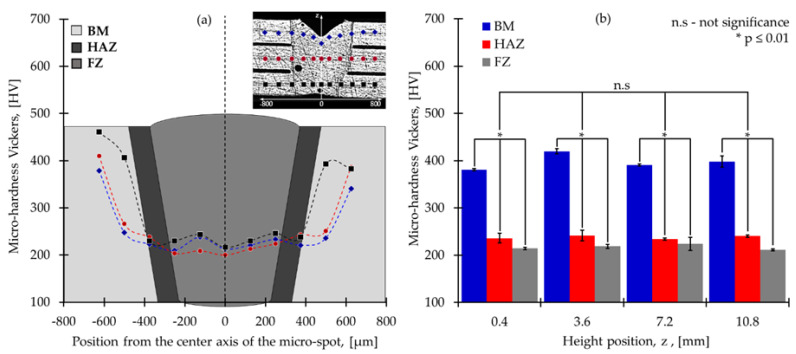
(**a**) Microhardness Vickers results in one spot and (**b**) microhardness results in one column of spots in all height scaffold.

Figure 15a shows the microhardness measurement results obtained from a micro spot welding. The material’s base hardness was 475 HV. Hardness is reduced in the HAZ and the fusion zone to 250 HV. This is due to the recrystallization of the product’s grains to the material’s fusion that takes place during laser welding. Figure 15b shows results obtained from Vickers microhardness at different height points in the scaffold. Obtained values for microhardness are 400 HV, 237 HV, and 215 HV for the base material, HAZ, and fusion zone, respectively. There was no significant difference between the different heights in the scaffold. This suggests a consistency in the mechanical properties obtained in the metallic scaffold. In contrast, there was a significant variation (i.e., *p*-value ≤0.01) between the harness in the base material, HAZ, and fusion zone.

## 4. Discussion

According to our results, the kerf width compensation in the laser cutting trajectories improved dimensional accuracy in the macro and micro size regimes. These results have a good agreement with the study performed by Aloke et al., which concluded that the kerf width has a major influence on the dimensional accuracy of the laser cutting process compared with thermal dilatation effects [34]. The maximum relative error found was 1.4% for all kerf width compensated samples up to 500 µm in length (see Figure 4). While the maximum relative error found was 2.7% for kerf width compensated samples below 500 µm of length. When medical devices such as scaffolds are manufactured, dimensional errors must be minimized in order to accomplish the bone ingrowth and to bio mimic the bone’s mechanical properties [17,18]. Additionally, the formation of dross and slag was observed in Figure 5m–p), which corresponds to samples manufactured in the micro size regimen (dimension lower than 500 µm). We attributed this phenomenon to the excessive accumulation of heat in a small area when the cutting path is performed. Further experiments must be performed to evaluate the influence of cutting paths on dimensional analysis.

AM involves promising processes for the fabrication of bone tissue-engineered porous scaffolds due to its versatility to produce complex structures. Additionally, bulk material can be processed without waste and lead-time consumption associated with traditional subtractive manufacturing methods [11]. However, limitations in the process capability are to be found, especially when the dimensional features range under 0.8 mm [35]. Wang et al. studied the process capability of selective laser melting (SLM) to produce porous structures with a pore size of 1000 µm and strut thickness of 300 µm with a maximum dimensional error of 11.7% in pore size. Their results demonstrated that powder adhesion is an inevitable effect of the process and affects the accuracy of the final pore’s dimensions [36]. We reported a maximum relative error in the X and Y axes of 1.4% and 19.6% for a length of 1000 µm for compensated and non-compensated geometries, respectively (Figure 4) for the calibration stage. While the manufactured scaffold resulted in a maximum relative error of ~5% (i.e., an average height of 11.564 mm) in the *Z*-axis due to the gaps obtained by the stacking process (Figure 7).

According to Garot et al. [4], the feedstock material cost of LPBF process is ~0.1$/g while the machine costs are between 40 k$ and 300 k$. LPBF processes are able to fabricate parts with a dimensional accuracy between 100 µm and 300 µm. Additionally, a recent study by Guarino et al., compared the fabrication of a metal washer produced by SLM and by laser cutting. Their results indicated that the cost per piece was € 0.63 to € 45.13 for laser cutting and SLM, respectively. The lifecycle analysis indicated that the environmental impact of SLM is 2.5 times higher than laser cutting. Guarino concluded that laser cutting is a more economical and ecological solution when compared with SLM [37]. In the present study, the dimensional lateral accuracy (X-Y) was under ± 5 µm. These results indicated higher accuracy in lateral directions (X-Y) than LPBF. For the proposed scaffold, the feedstock was 36 USD (43 sheets), the machine costs 219 k USD, and the installment required space was 4 m^3^. Additionally, the JS-LMSW process requires fixture costs of 1 k USD.

For the dimensions of the micro spots welds, the penetration depth (d) resulted in a narrower (w) than spot width when z-height is up to 2 mm (Figure 9). The morphology of all the micro spot welds is like a parabolic shape due to penetration depth which is larger than the welding spot; this suggests that the micro spot welds are created in a keyhole regimen [38]. Additionally, for visual inspection (Figure 7b), there is a change of shape at the scaffold’s base compared to the upper layers. It can be explained because of the less heat transferred on the *Z*-axis compared to the lateral X and Y axes. This effect was presented when HAZ was studied. Heat-affected zones resulted in a narrower (Figure 10b) bottom part of the spot weld compared with the lateral zone. Ventrella et al. reported a similar phenomenon in the laser seam welding; of AISI 304 metallic foils in an overlap configuration, where the heat-affected zone was wider in the top metallic foil than at the bottom. He explained that the gap between the metallic foils acted as a barrier and was the cause behind that energy dissipated in the lateral direction, which resulted in wider heat-affected zones [39]. This isolating effect was corroborated by Figure 10b, where the microstructure of the bottom sheet remained equal to the BM. This indicated that the amount of heat transferred to the bottom sheet was very low in comparison to the amount of heat transferred in the lateral directions. Additionally, some pores were observed in the fusion zone. These defects represent an area to improve the proposed process since these defects could have a negative influence on the metallic scaffold’s mechanical properties [40]. The largest shrinkages were found in the center zone of the scaffold; this suggests that the concentration of heat caused by the sheet stacking and the path welding strategy increased the shrinkage.

Furthermore, the microstructure observed in the micro spot welds is a result of the primary solidification mode of ferrite to austenite (FA mode). This mode of solidification is common in austenitic stainless steels whose CrEQ/NiEQ equivalent ratio is between 1.48 and 1.95 (i.e., AISI302 has a CrEQ/NiEQ = 1.8). As the cooling rates increase, the transformation from ferrite to austenite takes on different microstructures [32]. The microstructures identified in Figure 10d occurred at high cooling rates. Besides, when the cooling rate is very high, all the ferrite is transformed, and only austenite is present. This is consistent with the microstructure observed in the micro-spot welds since in the HAZ and the border of the FZ, more structures of the vermicular and lathy ferrite type were observed. However, as we approached the center of the fusion zone, we only found an austenite phase. This indicates extremely high cooling rates in the center of the weld due to the laser welding process. The EDS analysis indicates a higher concentration of precipitates in the HAZ; this occurs when the material is heated close to the solidus temperature, which causes supersaturation of the austenite matrix [32]. Additionally, HAZ showed a hardness of 237 HV, greater than the hardness of 215 HV presented in the fusion zone (see Figure 14a). These results suggest that the supersaturation of precipitates produced a hardening effect in the HAZ zone. Additionally, the base material did not present a grain thickening close to the HAZ; this is a common behavior in materials that come from cold work. However, due to the recrystallization of the material and due to the melting at the spot weld, softening occurred in the HAZ and fusion zones presenting lower values of hardness than the base material. Solati et al. [41] reported a reduction in the hardness results from the BM forward to the center of the FZ due to changes in microstructure in laser seam welds of AISI316L sheet of 0.6 mm of thickness. The microstructure observed in this study has also been observed in other stainless steels (i.e., AISI304, AISI316) seam welded by an Nd-YAG laser [42].

According to the bone mechanical properties found in literature listed in Table 5, the compression module obtained in the created scaffolds using the proposed methodology behaves similarly to that of the cortical bone, with a higher compressive yield and maximum stress [43]. These results prove that the developed scaffolds offer a wide safety window towards the stress shielding effect. While exceeding the mechanical properties of bone tissue, the unique architecture (porosity and morphology) provides a wide surface area for osteoblast migration, proliferation, and ultimately, surface and inner mineralization leading to full integration of the implant [44,45]. Further research will be performed to improve the scaffold’s design as well as to increase the porosity level to achieve a higher degree of resemblance to the mechanical properties like cortical bone. Another parameter that should be taken into consideration is the elastic modulus, which should behave similarly to that of the bone in order to ensure adequate load transfer, which has been widely studied in dental implants [46,47]. Additionally, further experiments must be centered in the application, specifically in vitro and in vivo tests. LPBF processes have demonstrated their capacity to manufacture medical applications with good results in clinical studies. For example, Pobloth et al. [48] reported the treatment of critical segmental bone defects in 19 patients with 3d-Ti mesh scaffolds fabricated by laser sintering. Radiographic analysis showed bone defect bridging in most of the cases after 28 months after implantation. However, according to Garot et al. [4], scaffold fabricated with AM techniques must be optimized to obtain an architecture with good mechanical and biological properties and implants tested clinically.

JS-LMSW allowed obtaining joined metallic sheets with the desired pore shape, size, and distribution. In comparison to the LPBF processes, JS-LMSW represents a smaller number of trajectories performed by the laser beam to obtain the same porous volume. Furthermore, joining the sheets by a matrix of discrete micro spot welds leads to a lower amount of energy used than the energy required for the fusion of the entire section and less heat dissipation which results in less probability of warping effects compared with LPBF [2]. Furthermore, the compression tests demonstrated that the LMSW avoids the inconsistent mechanical properties caused by no uniform heat dissipation [2] or porosities due to partial fusion [4], which are commonly presented in LPBF. In our study, the measured porosity resulted similarly to designed porosity (i.e., a relative error between 0.5% and 1.1%). The use of metallic sheets as feedstock eliminates the problems related to the removal of the entrapped powder and material wastage presented in LPBF [2]. However, further experiments must be performed to evaluate the presence of porosities and shrinkages in the spot weld as a way to avoid them and their effect on mechanical testing. The potential solutions are to change the shielding gas to N2, to reduce the free spaces between stacked sheets modifying the fixture, and to evaluate other values of laser intensity through the laser beam defocusing [49,50].

## 5. Conclusions

The novel fabrication of austenitic stainless steel scaffolds for bone tissue engineering applications was successfully achieved. The sheet lamination AM proposed methodology showed high consistency and a promising future in the tissue engineering field. This study drew the following conclusions:

- After the calibration study, the accuracy of the laser micro-cutting was 1.4% for all kerf width compensated samples of up to 500 µm (macro-shapes) of length and 2.7% for kerf width compensated samples below 500 µm (micro-shapes) of length. This indicated the capability of the methodology to produce geometries in the micro size regimen with high repeatability;
- The micro-spot welds presented a penetration depth between 704 and 736 µm, a width between 584 and 616 µm, and a HAZ between 25 and 34 µm. Shrinkages and pores were identified as the principal defects to mitigate in future investigations;
- The microstructures of vermicular and lathy ferrite observed in the FZ and HAZ regions of the spot welds indicated high cooling rates during the laser welding process. Additionally, these high cooling rates caused a supersaturation of precipitates in the HAZ regions. The high cooling rates and the gaps observed between sheets are probably the main cause of the shrinkage defects in the micro-spot welds;
- The fabricated scaffolds presented a compressive modulus between 13.03 GPa and 13.26 GPa. which are close to the cortical bone properties. Additionally, the scaffolds showed a measured porosity between 46%–47%, which is in good agreement with the conceptual design;
- The main reason for the failure of the scaffolds at compression tests was the sliding of the AISI302 sheets. This phenomenon occurs between the 0.1 and 0.2 strain deformation. The sliding of the sheets causes bending deformations, and the collapse initiates shearing cracks in the interfaces of the sheets;
- The microhardness obtained was 400 HV, 237 HV, and 215 HV for the base material, HAZ, and fusion zone, respectively. The recrystallization of the material due to the melting of the material caused a softening effect in the FZ and HAZ regions. The supersaturation precipitates caused a hardening effect in the HAZ region in comparison with the FZ. No significant difference was found between the microhardness measurement along with different height positions of the scaffold.

## Figures and Tables

**Table 1:** AISI316L bone scaffolds mechanical properties found in the literature.

Ref.	Process	Compressive Modulus (GPa)	Compressive Strength (MPa)	Measured Porosity (%)
[13]	SLS	26–43 GPa	20–32	50–46
[14]	SLS	1.58–6.64 GPa	15.5–52.8	58–28
[15]	SLM	0.15 GPa	3	87
[16]	Binder Jetting	0.26–3.06 GPa	6.3–545.5	50–27

**Table 2:** Process parameters for the laser micro-cutting and micro-spot welding operations.

	Laser Micro-Cutting	Laser Micro-Spot Welding
Average Laser Power, P_AVG_ (W)	170	220
Pulse frequency, f (Hz)	1100	-
Assist gas pressure, p_1_ (psi)	150	≈0
Feed rate, f (mm/min)	200	-
Exposure time, t (ms)	-	50
Shielding gas pressure, p_X_ (psi)	-	≈0
Shielding gas flux, f_X_ (L/min)	-	15

**Table 3:** Nominal dimensions of each sample for laser micro-cutting calibration.

	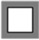 Column 1	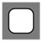 Column 2		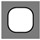 Column 3		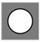 Column 4
	L (μm)	L (μm)	R (μm)	L (μm)	R (μm)	R (μm)
Row 1	3000	3000	750	3000	1000	1500
Row 2	2000	2000	500	2000	667	1000
Row 3	1000	1000	250	1000	333	500
Row 4	500	500	125	500	167	250
Row 5	250	250	63	250	83	125

**Table 4:** Results of the EDS analysis for the phases and precipitates.

	Fe	Cr	Ni	Mo	Si
γ	72	19	6.7	1.2	0.9
δ	75	20	4.5	-	-
P1	61	29	6.9	1.2	0.8
P2	48	19	30	0.7	1.2

Note: All values are % weight.

**Table 5:** Mechanical properties in compression of the human cortical bone and the fabricated scaffold.

	[15]	[13]	This Study
Material	Natural bone	AISI316L	AISI302
Measured porosity (%)	3–12	40–50	46–47
Compressive modulus (GPa)	14–28	26–46	13.03–13.26
Yielding stress (MPa)	120–175	-	550–554
Ultimate strength (MPa)	190–245	21–32	649–655
Fabrication method	-	SLS	JS-LMSW
